# Front-line paclitaxel and irinotecan combination chemotherapy in advanced non-small-cell lung cancer: a phase I–II trial

**DOI:** 10.1038/sj.bjc.6602827

**Published:** 2005-10-25

**Authors:** G P Stathopoulos, J Dimitroulis, D Antoniou, C Katis, D Tsavdaridis, O Armenaki, C Marosis, P Michalopoulou, T Grigoratou, J Stathopoulos

**Affiliations:** 1 First Oncology Deptartment, Errikos Dunant Hospital, Athens, Greece; 2 Sotiria Hospital, 6th Clinic, Greece; 3 Sotiria Hospital, 7th Clinic, Greece; 4 Thriacion Hospital, Greece; 5 IKA Hospital, Thessalonica, Greece; 6 Sotiria Hospital, 3rd Clinic, SOLCA Study Group, Greece; 7 Sotiria Hospital, 5th Clinic, SOLCA Study Group, Greece

**Keywords:** irinotecan, paclitaxel, non-small-cell lung cancer

## Abstract

Our purpose was to determine the efficacy of irinotecan plus paclitaxel administered on day 1, repeated every 2 weeks, in untreated patients with advanced or metastatic non-small-cell lung cancer (NSCLC). In total, 56 patients with inoperable or metastatic stage III and IV NSCLC with a histologically or cytologically confirmed diagnosis were enrolled. None of the patients had undergone prior chemotherapy or radiation therapy. Treatment involved irinotecan 125 mg m^−2^ and paclitaxel 135 mg m^−2^ administered on day 1 and repeated every 2 weeks for a planned number of nine cycles. With a standard dose of paclitaxel at 135 mg m^−2^, the dosage of irinotecan was escalated at four levels: 75, 100, 125 and 150 mg m^−2^; 125 mg m^−2^ was established as the maximum tolerated dose; this dosage was administered to 46 patients. A total of 52 patients (median age 65 years, range 38–77 years) were assessable for toxicity and survival and 46 for response rate. Out of 46 evaluable patients, 19 achieved partial response (41.3%), 17 had stable disease (37%) and 10 (21.7%) experienced disease progression. The median duration of response was 6 months (range 2–9+ months). The main adverse reactions were myelotoxicity (grades 3 and 4) in 10 (19.2%) patients and diarrhoea (grade 3) in four (7.7%) patients. Irinotecan combined with paclitaxel, administered every 2 weeks, appears to be an effective treatment for advanced-stage NSCLC.

Advanced-stage non-small-cell lung cancer (NSCLC) patients are still in need of new chemotherapy agents that will improve response rate and survival. New agents have been effectively established for other malignant tumours but very few have been applied in cases of NSCLC. One class of recent cytotoxic drugs are the camptothecins. Irinotecan (CPT-11) is a semisynthetic derivative of camptothecin, which produces a cytotoxic effect through interaction with and stabilisation of DNA/topoisomerase-I cleavable complexes by the active metabolite SN-38. The collision of the transcription apparatus and the stabilisation of the cleavable complex cause damage and lead to cell death (Douillard *et al*, 2000; Sargent *et al*, 2001). Irinotecan has been administered in colorectal cancer, mainly in combination with other agents in advanced disease (Wasserman *et al*, 1999; Saltz *et al*, 2000; Goldberg *et al*, 2004); it is also effective in pancreatic cancer (Rocha-Lima *et al*, 2002; Stathopoulos *et al*, 2003). The choice of irinotecan for gastrointestinal cancers is more or less empirical and use within clinical trials for other malignant tumours may show equal effectiveness. Existing data combining irinotecan with other agents such as cisplatin (Cardenal *et al*, 2003) show an effectiveness comparable to other established treatments in NSCLC (Negoro *et al*, 2003). For the present study, the second agent selected for combination treatment with irinotecan was paclitaxel, which has been tested as first-line combined treatment for advanced NSCLC (Chang *et al*, 1996; Greco and Hainsworth, 1997; Belani *et al*, 1999; Bonomi *et al*, 2000; Stathopoulos *et al*, 2004). *In vitro* combination studies of topoisomerase inhibitors with taxanes have shown conflicting results. In one preclinical study, the combination of a topoisomerase inhibitor in combination with paclitaxel demonstrated synergistic cytotoxicity (Chou *et al*, 1994). Another preclinical study has shown that this combination in human NSCLC cell lines is antagonistic (Kaufmann *et al*, 1996). One interpretation is that these interactions may vary, depending on the cell type studied and the sequence of drug administration (Adjei *et al*, 2000). It was suggested that this combination be tested in clinical trials.

In the present phase I–II trial, we combined paclitaxel with irinotecan, administering both agents on the same day, repeating treatment every 2 weeks. This biweekly (every 2 weeks) chemotherapy schedule was based on recent trials with proper dosage adjustment and a successful outcome (Cardenal *et al*, 2003; Negoro *et al*, 2003). Our objectives were to determine response rate, toxicity, and median and overall survival.

## PATIENTS AND METHODS

### Eligibility criteria

Eligibility for the study required the following: histologically or cytologically confirmed NSCLC, disease staging (operation for staging was permitted) and defined inoperable stage IIIB, IV, no prior chemotherapy or radiotherapy, bidimensionally measurable disease on physical examination, X-rays, computed tomography (CT), World Health Organisation (WHO) performance status 0–2, expected survival ⩾12 weeks, adequate bone marrow reserve (leucocyte count ⩾3500 *μ*l^−1^, platelet count ⩾100 000 *μ*l^−1^ and haemoglobin ⩾10 g dl^−1^), adequate renal function (serum creatinine ⩽1.5 mg dl^−1^) and liver function (serum bilirubin ⩽1.5 mg dl^−1^ and serum transaminases ⩽ three times the upper limit of normal (or ⩽ five times the upper limit of normal in cases of liver metastases]), and age ⩾18 years. In cases with central nervous system (CNS) involvement or any secondary malignancy, patients were excluded. This study was conducted with the approval of our institutional review board and all patients gave their informed consent before entering the study.

### Treatment plan

Paclitaxel and irinotecan were administered in different intensities: once weekly or every 3 weeks with adjusted doses. Paclitaxel was given biweekly (once every 2 weeks) at a dose of 135 mg m^−2^, in combination with a second agent, and toxicity was well-tolerated (Stathopoulos *et al*, 2004). The dose of irinotecan combined with another agent, administered on days 1 and 8, repeated every 21 days, varied from 60 to 110 mg m^−2^ (Kakolyris *et al*, 2000, 2001; Keresztes *et al*, 2004). In the present study, our intention was to conduct a phase I study by escalating the dosage of irinotecan while keeping paclitaxel at a standard dose. This was considered necessary since the dose of irinotecan had not been established when given once every 2 weeks in combination with another myelotoxic agent. On the basis of the previously mentioned data, we determined the dosages as follows: four levels of irinotecan starting at 75 mg m^−2^, increasing the dose by 25 mg m^−2^ and ending at a dose of 150 mg m^−2^. We intended to increase the latter to a higher dose if it was well tolerated. The dose of paclitaxel remained at 135 mg m^−2^, as this had been tested in a biweekly administration in combination with other myelotoxic agents such as carboplatin or navelbine (Stathopoulos *et al*, 2004). Table 1 shows dose escalation. When the maximum tolerated dose (MTD) was decided upon (detected or reached), it was given to the next recruited patients. Both drugs were infused: first, irinotecan in normal saline 500 cm^3^ for 90 min and then paclitaxel in a solution of 300 mol mixture of 5% dextrose and normal saline for a 3-h infusion. Premedication of 8 mg dexamethasone and 50 mg diphenhydramine was given 1 h before paclitaxel administration and repeated every 8 h for the first day and once daily on days 2 and 3. Ranitidine in a normal saline infusion was given at the beginning of the treatment. Ondansetron (8 mg) was also administered at the beginning and at the end of the infusions. The treatment plan was to repeat the drug administration every 2 weeks for nine cycles unless there was disease progression. In cases of myelotoxicity (neutropenia or thrombocytopenia), dose adjustments within a cycle were based on weekly absolute granulocyte and platelet counts and clinical assessment.

### Baseline and treatment assessments and evaluation

Before study entry, all patients underwent the following evaluations: physical examination, tumour evaluation or measurement, WHO performance status, ECG, full blood count, liver and kidney function tests and urinalysis. Staging was determined by chest and abdominal CT scans, bone scan and occasional magnetic resonance imaging. Blood count, blood urea and serum creatinine were measured before each treatment administration and 7 days after treatment. During the treatment period, radiologic tests were conducted after four courses, at the end of the study and after any course if the clinical signs were indicative of disease progression.

Response and toxicity were assessed using standard WHO criteria. Complete response (CR) was considered to be the complete disappearance of any sign of demonstrable disease, partial response (PR) as ⩾50% reduction of measurable disease and stable disease (SD) as a <50% reduction or a <25% increase in the sum of the products of the two perpendicular diameters of all measured lesions and the appearance of no new lesions for 8 weeks. Progressive disease (PD) was defined as an increase in the product of the two perpendicular diameters of any measurable lesion by 25% over the size at the time of maximum regression and the appearance of new areas of malignant disease. The duration of response was measured from the documentation of response (CR or PR) to PD. The time to tumour progression (TTP) was measured from the time of the first dose administration to disease progression. The determination of objective response on computed tomography was performed by two independent radiologists and two experienced oncologists.

### Statistical design

This was an expected two-step phase II study after the integration of the phase I study. According to the trial design, 30 patients were to be enrolled during the first part of the study, and if an objective response rate of <15% was achieved, the treatment would have been abandoned; otherwise, 20 additional patients were to be enrolled. The primary end point of the study was the efficacy of the regimen and the secondary end points were overall survival (OS) and tolerance. Overall survival was calculated from the day of enrolment until death. The median probability of survival and median TTP were estimated by the Kaplan–Meier method; confidence intervals (CIs) for response rates were calculated using methods for the exact binomial CI.

## RESULTS

Of the 56 intent-to-treat patients with advanced inoperable NSCLC, enrolled in the study between October 2003 and February 2005, 52 (92.9%) were evaluable for toxicity and survival. A total of 46 patients were evaluated for response rate. Three patients at dose escalation level 1 and three at level 2 were excluded as the dosage they received was inferior to the MTD. Four out of 56 patients were nonevaluable: two refused to continue after the first infusion and the other two were excluded due to cardiac disease nonrelated to the toxicity of the treatment. A total of 47 (83.9%) patients were male and nine (16.1%) were female (median age 65 years, range 38–77 years), and 25 and 31 were stage IIIB and IV, respectively. The patients' characteristics at baseline are shown in Table 2. Histology showed 21 squamous cell type, 19 adenocarcinoma, one large cell type and 15 undifferentiated or nondefined by cytological examination.

### Compliance with treatment–dose intensity

**Figure 1:**
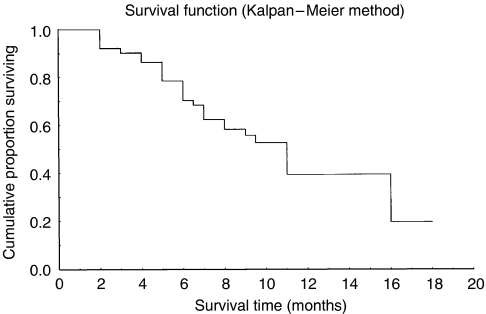
Survival distribution estimation (Kaplan–Meier method).

In total, 318 chemotherapy cycles were administered with a median of 6.63 cycles (range 2–9) per patient. The median interval between cycles was 15 days (range 15–22 days). Expected dose intensity for paclitaxel was 67.5 mg m^−2^ week^−1^ (mean dose intensity 66.2 mg m^−2^ week^−1^); the 95% CI was 65.1–67.2 mg m^−2^ week^−1^ (median dose intensity 67.5 mg m^−2^ week^−1^, range 54–67.5 mg m^−2^ week^−1^). The expected dose intensity for irinotecan was 60 mg m^−2^ week^−1^ (mean dose intensity 59 mg m^−2^ week^−1^); the 95% CI was 58.3–59.8 mg m^−2^ week^−1^ (median 60 mg m^−2^ week^−1^, range 50–60 mg m^−2^ week^−1^). In four patients, four cycles (once per patient) were delayed by 1 week. Three out of six patients at level four presented with grade 4 neutropenia and four out of six had grade 2 or 3 diarrhoea. In patients at dosage levels 1 and 2, grade 1 neutropenia and grade 1 or 2 diarrhoea were observed in two out of six patients. Of the 46 patients included in the phase II study (at dosage level 3), in two the dosage of both cytotoxic agents was reduced by 25% (after the third cycle in one, and after the fourth in the other). Treatment delay or dose reduction was due to grade 3 or 4 neutropenia in five cases and to hepatotoxicity (transaminasemia reversible) in one case. At the time of this analysis, 25 patients were still alive (48.1%) with a median follow-up time of 10 months (range 5–16 months). The Kaplan–Meier method was used for survival distribution estimation (Figure 1).

### Response rate and survival

An objective response rate was observed in 19 out of the 46 evaluable patients (41.3%), with a median duration of response of 6 months (range 2–9+). The response rate and duration by stage are shown in Table 3. The responders were PR and there were no CRs. A total of 17 patients showed SD (37%) and 10 patients had disease progression (21.7%). The median TTP was 6 months (range 2–11+ months).

### Toxicity

In the 15 patients included in the phase I escalation testing, no serious toxicity was observed at levels 1 and 2. Haematologic toxicity was, in general, acceptable at the level 3 dose (125 mg m^−2^ of irinotecan and 135 mg m^−2^ of paclitaxel). With regard to the level 4 dosage, 50% of the patients presented with grade 4 neutropenia and grade 3 diarrhoea. Serious neutropenia was observed in 10 patients (19.2%): eight with grade 3 (15.4%) and two with grade 4 (3.8%). Febrile neutropenia was seen in three (5.8%) of these patients. All of the above patients were treated with haemopoietic growth factor, given on days 6–8 following the previous treatment cycle. Anaemia was common (23 patients), but only four had grade 3 (7.7%). Grades 1 and 2 thrombocytopenia were uncommon (five patients, 9.6%) (Table 4). Mild nonhaematologic toxicity was observed in the majority of the cases: grade 3 toxicity with diarrhoea in four patients (7.7%), alopecia in four patients (7.7%), fatigue in one patient (1.9%) and allergy in one patient (1.9%). Nonhaematologic toxicity is shown in Table 5.

## DISCUSSION

This trial was planned in order to test a new combination as first-line treatment in advanced and inoperable NSCLC. Paclitaxel, one of the two agents, is an established drug in a combined modality for NSCLC patients. Much data exist where paclitaxel has been administered with different agents (mainly cisplatin or carboplatin) (Akerley *et al*, 1997; Belani *et al*, 1999; Frasci *et al*, 1999; Bonomi *et al*, 2000; Johnson *et al*, 2004) as first-line treatment in advanced NSCLC. Irinotecan has been administered in NSCLC with agents other than paclitaxel either as second-line (Kakolyris *et al*, 2000, 2001; Keresztes *et al*, 2004; Ohyanagi *et al*, 2004) or as front-line treatment with cisplatin (Cardenal *et al*, 2003; Negoro *et al*, 2003). It was our hope to further determine the cytotoxic value and effectiveness of irinotecan in NSCLC. In recent years, several new agents have been introduced for the treatment of advanced NSCLC. Some have an established effectiveness, such as taxanes, including docetaxel (Fossella *et al*, 2000; Georgoulias *et al*, 2001), gemcitabine (Crino *et al*, 1997, 1999) and vinorelbine (Le Chevalier *et al*, 1994; Kouroussis *et al*, 1998), as well as paclitaxel. Although other new tested agents exist, their effectiveness needs to be confirmed: teniposide, premetrexed and irinotecan are already in use in NSCLC clinical trials. Teniposide in combination with cisplatin produced a reasonably high response rate (41%) with a 1-year survival of 43% (Giaccone *et al*, 1998). Premetrexed was initially tested as second-line treatment and produced a 23.3% response rate, with quite high but acceptable toxicity (Rusthoven *et al*, 1999). Then, premetrexed was combined with cisplatin as first-line treatment and the combination was effective with a 45.8% response rate, 8.5 months median survival and 40% 1-year survival (Shepherd *et al*, 2001). Irinotecan may prove to be a promising agent but further confirmatory trials are required. The existing data are mainly related to second-line treatment using irinotecan on days 1 and 8 and cisplatin on day 8 repeated every 21 days with doses of 100–110 and 80 mg m^−2^, respectively: PR was 16.7% and the main toxicity was grade 3–4 neutropenia (18%) and diarrhoea (29%) (Kakolyris *et al*, 2000). Another trial with the same agents and dosages produced similar toxicity and a 20% response rate, which cannot be ignored as a second-line chemotherapy following a docetaxel-based front-line regimen (Kakolyris *et al*, 2001). In another randomised study, irinotecan was combined with docetaxel *vs* irinotecan with gemcitabine, also as second-line treatment with or without celecoxib, in patients with NSCLC. The dose of irinotecan was 60 mg m^−2^ when combined with docetaxel and 100 mg m^−2^ when combined with gemcitabine, administered on days 1 and 8, with both agents repeated every 21 days. The response rate was low with a PR of 4–8.5%; toxicity was mainly diarrhoea in 13–28% of the patients, neutropenia in 20–26% and thrombocytopenia in up to 34% (the latter was observed only in the irinotecan–gemcitabine combination) (Keresztes *et al*, 2004). The combination of irinotecan with gemcitabine was the object of another trial as second-line treatment for refractory or relapsed NSCLC. In this latter study, there was a difference, in comparison to the other aforementioned studies, with respect to the dosage of irinotecan and to treatment intensity: irinotecan 150 mg m^−2^ and gemcitabine 1000 mg m^−2^ were given on days 1 and 15, repeated on day 28. Haematologic toxicity and diarrhoea were lower at 7.4–14.8 and 3.7%, respectively. The lower toxicity might have been due to eliminating treatment on the 8th day. Response was 18.5% (Ohyanagi *et al*, 2004). All of the above trials were preliminary with a small number of patients. In our study, where the drug administration was also every 2 weeks, the 150 mg m^−2^ dose during phase I, dosage level 4 (six patients), toxicity related to grade 4 neutropenia was detected in three out of six (50%) patients and grade 3 diarrhoea in three out of six (50%) patients. The difference from the previous trial might be due to the combined second agent, which in our study was paclitaxel.

Two other trials have tested irinotecan as first-line chemotherapy. One study combined irinotecan with cisplatin and both agents were given on day 1 and repeated every 3 weeks. The dosage of irinotecan was 200 mg m^−2^ and cisplatin 80 mg m^−2^. These investigators achieved a 34.2% PR rate and the toxicity, again, was mainly diarrhoea (29%) and febrile neutropenia (14%); the 1-year survival was 31%, and the median survival was 8.2 months (Cardenal *et al*, 2003). The other trial with the irinotecan and cisplatin combination as first-line treatment was a randomised phase III study, comparing this regimen with the cisplatin and vindesine combination, and there was also a third arm with irinotecan alone. The authors concluded that there was no statistically significant difference among the three arms with respect to response rate and survival. In the irinotecan–cisplatin arm, the response rate was higher (50%) than that of the other arms, but the toxicity was also the highest (diarrhoea 12–15%). Irinotecan administration was on days 1, 8 and 15, every 4 weeks at a dose of 60 mg m^−2^ (Negoro *et al*, 2003).

In a phase I trial, CPT-11 (irinotecan) was one of the three agents used in combination with carboplatin and paclitaxel in NSCLC patients with advanced disease. The treatment was administered once every 3 weeks. Toxicity was high with 50% myelotoxicity and 19% febrile neutropenia. These authors suggest the MTD as CPT-11 100 mg m^−2^, carboplatin 5 AUC and paclitaxel 175 mg m^−2^. The response rate of 39% is not higher than that of other treatments with two agents, but it is quite toxic. The median survival of 11 months was satisfactory, but it is inconclusive as to whether or not the addition of CPT-11 to a well-tested two-drug combination (carboplatin–paclitaxel) affected the efficacy; it is, however, more or less certain that it increased the toxicity (Socinski *et al*, 2001).

The same combination of CPT-11 100 mg m^−2^, carboplatin 5 AUC and paclitaxel 175 mg m^−2^ once every 3 weeks was given in a phase II study by the same group of investigators who consider that this regimen can be safely administered in NSCLC patients. There was, however, 78% neutropenia. Responsiveness was 32% and SD 55%. The median survival was quite remarkable at 12.5 months (Socinski *et al*, 2002).

Another phase I study in NSCLC patients, by a Japanese group of researchers, combined paclitaxel (day 1) with CPT-11 (days 1, 8 and 15) in a 4-week cycle; a dosage of paclitaxel 210 mg m^−2^ and CPT-11 50 mg m^−2^ was defined as dose-limiting toxicity (DLT). The MTD was 50 mg m^−2^ for CPT-11 and 180 mg m^−2^ for paclitaxel. The repetition of CPT-11 on days 8 and 15 may not have added to the efficacy but only increased the toxicity (Kasai *et al*, 2002).

Similar results are given in another phase I–II study where paclitaxel and CPT-11 were combined in NSCLC patients. The treatment was performed every 2 weeks, as in our study, and the recommended dose was 160 mg m^−2^ for paclitaxel and 60 mg m^−2^ for CPT-11 – a higher dose of paclitaxel and a much lower dose of CPT-11 as compared to ours. Although the number of patients was only 24, it is interesting to note that the response rate was 58.3% and the 1-year survival was 54.2% (Yamada *et al*, 2004).

A phase I study with the paclitaxel and irinotecan combination was different from ours and the aforementioned trials, in that the agents were administered on days 1 and 8 and repeated every 3 weeks. These authors concentrated on the pharmacokinetic analysis and suggested that the MTD was 40 mg m^−2^ for irinotecan and 50 mg m^−2^ for paclitaxel. Only nine patients were tested and the effectiveness described was SD (Hotta *et al*, 2004).

Based on the above, concerning the use of irinotecan in NSCLC, one can assume that (a) this agent is an effective product for this disease, particularly when combined with another cytotoxic drug, and (b) it is nonconclusive as to whether the treatment schedule should be weekly, biweekly (every 2 weeks) or every 3 weeks. Our study produced a response rate of 41.3%, which is higher than that reported in most of the studies with irinotecan and is comparable to other studies with established chemotherapy schedules. Serious neutropenia and diarrhoea in the present trial were approximately 20%. Comparing the toxicity in irinotecan trials, it appears that it is lower and more acceptable when the schedule is repeated every 2 weeks, as shown in our study and also in the other aforementioned study using a biweekly schedule (Ohyanagi *et al*, 2004). It appears that in eliminating the 8th-day repetition of drug administration, the response rate is not reduced. Whether the biweekly dose of irinotecan should be 125 mg m^−2^ as in our trial or 150 mg m^−2^ as in the other trial (Ohyanagi *et al*, 2004) is debatable, since toxicity and response in both trials were similar. Our lower dose of irinotecan was decided upon because it was combined with paclitaxel, a more myelotoxic agent than cisplatin, which was the agent used in the other study.

Irinotecan combined with paclitaxel as front-line chemotherapy in advanced or metastatic NSCLC is an effective treatment which, when given every 2 weeks, produces well-tolerated toxicity.

## Figures and Tables

**Table 1:** Dose escalation scheme

**Dose level**	**Irinotecan (mg m^−2^)**	**Paclitaxel (mg m^−2^)**	**No. of patients**	**Courses**
1	75	135	3	12
2	100	135	3	12
3	125	135	3+43^a^	282
4	150	135	6	12
				
Total				318

a A total of 43 patients were in the phase II part of the study, at this dose level.

**Table 2:** Patients' characteristics at baseline

**Characteristic**	**No. of patients**	**%**
No. of patients enrolled	56	
No. of patients assessable	52	92.9
		
*Age (years)*		
Median, 65		
Range, 38–77		
		
*Gender*		
Male	47	83.9
Female	9	16.1
		
*Performance status (WHO)*		
0	12	21.4
1	33	58.9
2	11	19.6
		
*Stage of disease*		
IIIB	25	44.6
IV	31	55.4
Total	56	100
		
*Histologic type*		
Squamous	21	37.5
Adenocarcinoma	19	33.9
Large cell	1	1.8
Undifferentiated	15	26.8
Total	56	100
		
*Tumour differentiation*		
Well differentiated	2	3.6
Moderately differentiated	18	32.1
Poorly differentiated	36	64.3
		
*Metastatic site*		
Bone	13	41.9
Liver	10	32.3
Lung	4	12.9
Adrenal	2	6.5
Brain	1	3.2
Pancreas	1	3.2
Total	31	

**Table 3:** Response rate and duration^a^ by stage

	**PR**	**SD**	**PD**	**Total**
**Disease stage**	***n* (%)**	***n* (%)**	***n* (%)**	***n* (%)**
IIIB	11 (23.9)	9 (19.6)	5 (10.9)	25 (54.3)
IV	8 (17.4)	8 (17.4)	5 (10.9)	21 (42.6)
Total	19 (41.3)	17 (37.0)	10 (21.7)	46 (100)

a Duration of response: median 6 months (range 2–9+ months).

PR=partial response; SD=stable disease; CR=complete response.

**Table 4:** Haematological toxicity

	**Grade 1**	**Grade 2**	**Grade 3**	**Grade 4**
	***n* (%)**	***n* (%)**	***n* (%)**	***n* (%)**
Neutropenia^a^	3 (5.8)	13 (25)	8 (15.4)	2 (3.8)
Anaemia	17 (32.7)	2 (3.8)	4 (7.7)	—
Thrombocytopenia	2 (3.8)	3 (5.8)	—	—

a Febrile neutropenia in three patients.

**Table 5:** Nonhaematologic toxicity

	**Grade 1**	**Grade 2**	**Grade 3**
	***n* (%)**	***n* (%)**	***n* (%)**
Nausea/vomiting	7 (13.5)	—	—
Diarrhoea	12 (23.1)	4 (7.7)	4 (7.7)
Alopecia	17 (32.7)	6 (11.5)	4 (7.7)
Fatigue	8 (15.4)	—	1 (1.9)
Allergy	5 (9.6)	1 (1.9)	1 (1.9)
Neurotoxicity	4 (7.7)	1 (1.9)	—
Myalgia	6 (11.5)	—	—
Mucositis	5 (9.6)	—	—
Cardiotoxicity	2 (3.8)	—	—
Nephrotoxicity	1 (1.9)	—	—
Hepatotoxicity	—	—	1 (1.9)
